# CCL21/CCR7 Enhances the Proliferation, Migration, and Invasion of Human Bladder Cancer T24 Cells

**DOI:** 10.1371/journal.pone.0119506

**Published:** 2015-03-23

**Authors:** Miao Mo, Mi Zhou, Lu Wang, Lin Qi, Kehua Zhou, Long-Fei Liu, Zhi Chen, Xiong-Bing Zu

**Affiliations:** 1 Department of Urology, Xiangya Hospital, Central South University, Changsha 410008, Hunan, P.R. China; 2 Department of Urology, Zhejiang Provincial People's Hospital, Hangzhou, Zhejiang 310014, P.R. China; 3 Health Management Center, Xiangya Hospital, Central South University, Changsha 410008, Hunan, P.R. China; 4 Department of Health Care Studies, Daemen College, 4380 Main Street, Amherst, NY 14226, United States of America; Faculty of Medicine & Health Sciences, UNITED ARAB EMIRATES

## Abstract

**Objective:**

To investigate the effects of CCL21/CCR7 on the proliferation, migration, and invasion of T24 cells and the possible associated mechanisms: expression of MMP-2 and MMP-9, and regulation of BCL-2 and BAX proteins.

**Methods:**

T24 cells received corresponding treatments including vehicle control, antibody (20ng/mL CCR7 antibody and 50 ng/ml CCL21), and 50, 100, and 200 ng/ml CCL21. Proliferation was evaluated by MTT assay; cell migration and invasion were assayed using a transwell chamber. Cell apoptosis was induced by Adriamycin (ADM). The rate of cell apoptosis was examined by flow cytometry using annexin V-FITC/PI staining. Western-blot was used to analyze MMP-2 and MMP-9 and BCL-2 and BAX proteins.

**Results:**

CCL21 promoted T24 cell proliferation in concentration-dependent manner with that 200 ng/mL induced the largest amount of proliferation. Significant differences of cell migration were found between CCL21treatment groups and the control group in both the migration and invasion studies (P < 0.001 for all). The expressions of MMP-2 and MMP-9 proteins were significantly increased after CCL21 treatment (p < 0.05 for all). Protein expression of Bcl-21 follows an ascending trend while the expression of Bax follows a descending trend as the concentration of CCL21 increases. No difference was found between the control group and antibody group for all assessments.

**Conclusion:**

CCL21/CCR7 promoted T24 cell proliferation and enhanced its migration and invasion via the increased expression of MMP-2 and MMP-9. CCL21/CCR7 had antiapoptotic activities on T24 cells via regulation of Bcl-2 and Bax proteins. CCL21/CCR7 may promote bladder cancer development and metastasis.

## Introduction

Bladder cancer is one of the most common types of adult cancer. In 2008 alone, 386,000 patients were diagnosed with bladder cancer which resulted in 150,200 deaths worldwide based on global statistics[1]. Metastasis is not only a hallmark of bladder cancer, but also the cause of mortality[1]. However, the pathophysiology of bladder cancer metastasis remains unclear. Chemokines, a superfamily of small secreted peptides characterized by their ability to induce leukocyte migration, together with their receptors have been found to be involved in the migration of cells of the lymphoid system, thus may affect cancer development and progression[2–4]. CCL21, an important chemokine of the CCL family, is one of the only two ligands (the other one is CCL19) for CCR7.[5] CCL21 is produced by fibroblastic reticular cells of the T-cell rich area in human and high endothelial venules in mice. [6] CCR7 is expressed by various types of lymphocytes including naive B and T cells, semimature and mature dendritic cells, and T regulatory cells[5]. In addition, the expression of CCR7 has been reported to promote cancer cell metastasis to lymph nodes in nonsmall cell lung cancer[7], breast cancer[8], squamous cell carcinoma of head and neck cancer [9], colorectal cancer[10], prostate cancer [11], esophageal squamous cell cancer[12] and gastric cancer[13]. Therefore, CCR7 and its ligand (s) may participate in the proliferation, progression, and metastasis of cancer cells of various organ origins[7–13]. We also found that CCR7 was involved in the development and progression of bladder cancer (unpublished data).

Physiologically, CCL21/CCR7 plays important roles in homing of immune cells, lymph-node homing and positioning, immunity and peripheral tolerance, development and function of T regulatory cells, and autoimmunity and lymphoid neogensis[5]. Various studies have confirmed the roles of CCL21/CCR7 in tumor development and progression[14–17]. For example, CCL21/CCR7 promotes G2/M phase progression and prevents apoptosis via the ERK pathway in human non-small cell lung cancer[15, 16], facilitates the progression of pancreatic cancer via induction of angiogenesis and lymphangiogenesis[14], regulates matrix metalloproteinase-9 (MMP-9) in human colon cancer metastasis[18], and upregulates MMP-9 in B-cell chronic lymphocytic leukemia cell migration and invasion [17]. Furthermore, CCL21/CCR7 was also found to promote cancer cell migration into microlymphatic vessels in breast cancer[19], pancreatic tumor[20], lung adenocarcinoma [21], and esophageal squamous cell carcinoma[22]. However, the possible role of CCL21/CCR7 in bladder cancer development and progression remains unclear. T24 cells are derived from transitional cell carcinoma of human urinary bladder and have been extensively utilized for the study of bladder cancer[23].

The purposes of the present study were to investigate the effects of CCL21/CCR7 on the proliferation, migration, and invasion of T24 cells and the possible associated mechanisms: expression of MMP-2 and MMP-9, and regulation of BCL-2 and BAX proteins.

## Materials and Methods

This study obtained ethics approval from the ethics committee at Xiangya Hospital, Central South University, Changsha, Hunan Province, China.

### Reagents and cell line

CCL21 recombinant human protein was purchased from Perprotech (Rocky Hill, NJ, USA) and polyclonal rabbit anti-human CCR7 antibody was purchased from Wuhan Boster Biological Engineering Co., Ltd. (Wuhan, China). Protease inhibitor Phenylmethylsulfonyl fluoride (PMSF) was purchased from Roche Diagnostics (Indianapolis, IN, USA). MTT and Dimethyl sulfoxide (DMSO) was purchased from Hufeng Chemical Co., Ltd. (Shanghai, China). Annexin V-FITC apoptosis detection kit was purchased from Beyotime Biotechnology (Shanghai, China).

The human bladder cancer T24 cell line was purchased from the Shanghai Institutes for Biological Sciences, the Chinese Academy of Sciences (Shanghai, China). The cells were cultured at 37°C, 5% CO_2_, in medium of DMEM (GIBCO, USA) containing 10% fetal bovine serum, 100 U/mL penicillin, and 100 U/mL streptomycin.

### Cell culture and treatment

T24 cells were treated for 48 hours prior to MTT assay, migration and invasion assays, and Western blot analysis and 24 hours prior to flow cytometry study. For each experiment, there were five groups consisting of Group 1 (control group) which was treated with serum free DMEM medium, Group 2 (antibody group) which first received pretreatment of 20 ng/mL of polyclonal rabbit anti-human CCR7 antibody for 4 h then 50 ng/ml of CCL21, Group 3 which received 50 ng/ml of CCL21, Group 4 which received 100 ng/ml of CCL21, and Group 5 which received 200 ng/ml of CCL21. The experiments of MTT assay, flow cytometry, and Western blot were repeated three times (n = 3), and cell migration and invasion were repeated four times (n = 4).

### MTT assay for the proliferation of T24 cells

Proliferation of T24 cells was evaluated by MTT assay. In brief, cells were treated correspondingly in each group for 48 hours. Following treatment, the medium was removed and the cells were incubated with 5mg/mL of MTT solution (20μl). After incubation for 4 h at 37°C and 5% CO2, the supernatant was removed and formation of formazan was measured at 490 nm with Gel Documentation & Analysis System set (Liuyi Factory, Beijing, China).

### Migration and invasion assays

Cell migration and invasion were assayed using a transwell chamber (EMD, Millipore, USA). For the invasion assay, a transwell chamber was placed into a 24-well plate and was coated with 30 μl Matrigel (Franklin Lakes, USA) and was incubated for one hour at 37°C. In both transwell assays, cells, after 48 hours' corresponding treatment, were trypsinized and seeded in chambers at the density of 8 × 10^4^ cells per well, and 500 μl of serum free DMEM medium was added to the lower chamber. Migrated cells were fixed with 100% Matrigel (BD, Franklin Lakes, USA) methanol for 30 min after 24 hours, and non-migrated Cells were removed by cotton swabs. Finally, cells on the bottom surface of the membrane were stained with hematoxylin and eosin for 20 min. Optical microscope (200X, Olympus, Tokyo, Japan) was used to count the number of cells in five random fields of view; mean cell number was calculated for each group.

### Flow cytometry for apoptosis

Cell apoptosis was induced by Adiamycin (ADM). The rate of apoptosis of T24 cells was examined by flow cytometry using annexin V-FITC/PI staining. Briefly, T24 cells were treated as above in each group for 24 h; cells were then incubated with ADM (0.1mg/ml) for additional 48 h. Then cells were harvested, washed and resuspended in PBS. Apoptotic cell death was measured by double staining annexin V-FITC and PI using the annexin V-FITC apoptosis detection kit (Beyotime Biotechnology, Shanghai, China) as per the manufacturer’s instructions. Flow cytometric analysis was performed immediately after staining. Data acquisition and analysis were performed by flow cytometry using the Cell Quest software.

### Western blot analysis

T24 cells were placed into 75 mL culture vials and were incubated at 37°C for 24 h and then treated correspondingly in each group for 48h incubation. Suspensions were washed with cold PBS. The cells were then incubated in ice-cold RIPA buffer [1 M Tris (pH 7.4), 5 M NaCl, 0.5 M EDTA (pH 8.0), 10% SDS, 10% DOS, and 10% NP40] with fresh protease inhibitor PMSF over ice for 20 min. The cells were scraped and the lysate was collected in an Eppendorff tube and centrifugated at 10,000 rpm at 4°C for 10 min, and the supernatants were collected, aliquoted, and stored at -20°C for future use.

Dilutions of BCL-2/BAX (12%) and MMP-2/MMP-9 (7.5%) were used for the study. Proteins (20 μg) were loaded in 5–15% SDS-PAGE gels and transferred onto a nitrocellulose membrane (Amersham Biosciences, USA). The membranes were soaked in blocking buffer (5% skimmed milk) under room temperature for 2 h. The blots were washed with PBS (with 0.1% BSA). To probe for MMP-2, MMP-9, BCL-2, Bax, and β-actin, the membranes were incubated 2h under room temperature with relevant antibodies, followed by appropriate HRP conjugated secondary antibodies and ECL detection. Gel Documentation & Analysis System set (Liuyi Factory, Beijing, China) was used for picture capture and analysis of Optical density (OD) values (490nm).

### Statistical analysis

Statistical analysis was performed using the SPSS 18.0 statistical software. Quantitative data were expressed as the mean ± SD. One-way analysis of variance with Fishers LSD test was used for group comparisons. The significance level was set at p-value less than 0.05.

## Results

### Effects of CCL21/CCR7 on the proliferation of T24 cells

The effects of CCL21/CCR7 on T24 cells as represented by OD values are presented in Table 1. OD values were 0.211 ± 0.013 in the control group, 0.216 ± 0.011 in the antibody group (p>0.05 as compared with control), 0.248 ± 0.006 in 50 ng/ml of CCRL21 (P < 0.05 as compared with control), 0.290 ± 0.004 for 100 ng/ml of CCRL21 (P < 0.01 as compared with control), and 0.341 ± 0.012 for 200 ng/mL of CCRL21 (P < 0.001 as compared with control). CCL21 promoted T24 cell proliferation in concentration-dependent manner with 200 ng/mL induced the largest amount of proliferation.

**Table 1:** Effect of CCL21 on T24 cell proliferation.

Groups	OD value	P-values
Control (DMEM medium)	0.211 ± 0.013	—
Anti-CCR7 Ab + CCL21 50 ng/mL	0.216 ± 0.011	P > 0.05
50ng/mL Group	0.248 ± 0.006	P < 0.05
CCL21 100 ng/mL	0.290 ± 0.004	P < 0.01
CCL21 200 ng/mL	0.341 ± 0.012	P < 0.001

Note: The OD values present Mean ± SD from 3 independent experiments.

### Effects of CCL21/CCR7 on the migration and invasion of T24 cells

**Fig 1:**
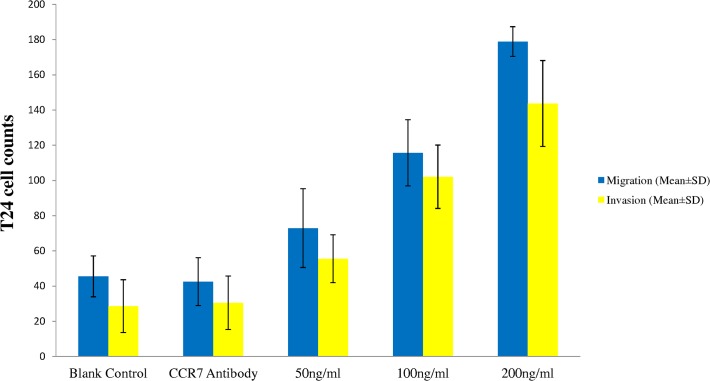
Effect of CCL21 with or without CCR7 antibody on cell migration and invasion of T24 cells. The assay was performed using a transwell chamber coated with 30 μl of Matrigel mixture solution with five different groups consisting of vehicle control, CCR7 antibody 20 ng/ml plus CCL21 50 ng/ml, CCL21 50 ng/ml, CCL21 100 ng/ml, and CCL21 200 ng/ml. The data present Mean ± SD from four independent experiments.

The results of CCL21/CCR7 on the migration and invasion of T24 cells are presented in Fig. 1. Cell counts for the migration and invasion studies are as the following: 45.5±11.6 and 28.6±15.0 for the control group, 42.5±13.6 and 30.5±15.2 for the antibody group, 72.9±22.4 and 55.5±13.6 for CCRL21 100 ng/mL group, 115.7±18.8 and 102.1±18.0 for CCRL21 150 ng/mL group, and 178.9±8.4 and 143.7±24.4 for CCRL21 200 ng/mL group. Significant differences of cell migration were found between either of the three CCL21treatment groups and the control group in both the migration and invasion studies (P < 0.001 for all); but, no difference was found between the control group and the antibody group in either the migration study or the invasion study (P > 0.05 for both). The effect of CCL21/CCR7 treatment on T24 cell migration is concentration-dependent with the highest concentration of CCL21 (200 ng/ml) produced the largest number of cells of migration/invasion. In general, smaller numbers of T24 cells were found in the invasion study than that of the corresponding migration study.

### Effects of CCL21/CCR7 on the expression of MMP-2 and MMP-9 in T24 cells

**Fig 2:**
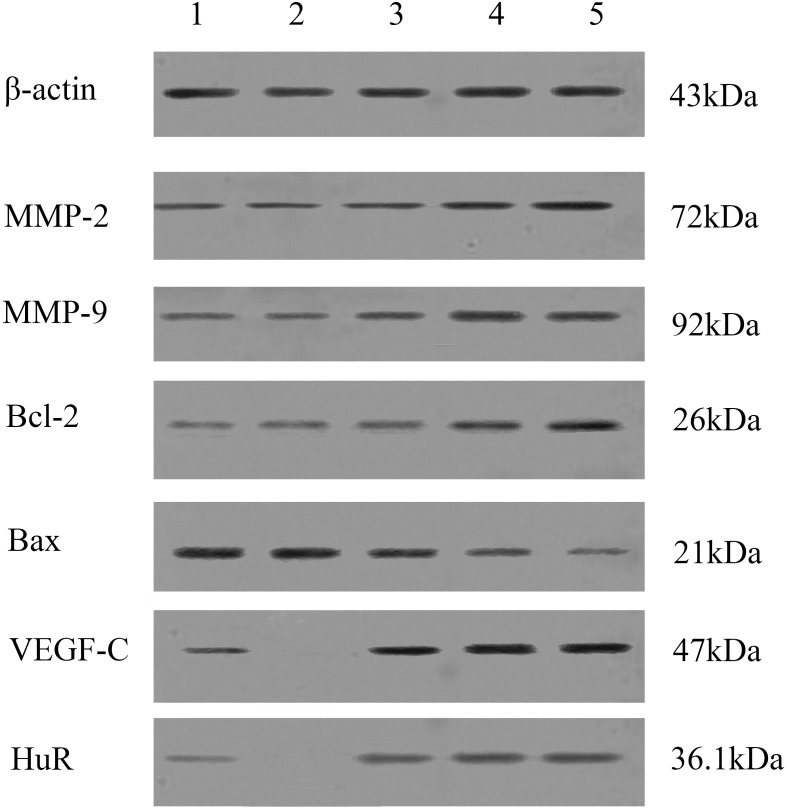
Effects of CCL21 with or without CCR7 antibody on the expression of MMP-2, MMP-9, Bcl-2, Bax, VEGF-C, and HuR by Western blot analysis. Lane 1: T24 cells treated with vehicle control; Lane 2: T24 cells treated with CCR7 antibody 20 ng/ml plus CCL21 50 ng/ml; Lane 3: T24 cells treated with 50 ng/ml CCL21; Lane 4: T24 cells treated with 100 ng/ml CCL21; and Lane 5: T24 cells treated with 200 ng/ml CCL21. Cell lysates were prepared and run on 5–15% SDS-PAGE gels following by a Western blot analysis. Beta-actin was used as a loading control. The blots shown are representative of three independent experiments.

Western blot results are presented in Fig. 2; quantitative measurements of MMP-2 and MMP-9 are presented in Table 2. Significant differences were found in the expression of MMP-2 and MMP-9 among the five groups (p < 0.05). As compared to the control group, the expressions of MMP-2 and MMP-9 proteins were significantly increased after three different concentrations of CCL21 treatment (p < 0.05 for all). However, no difference was found in MMP-2 or MMP-9 between the control group and the antibody group (p > 0.05 for both).

**Table 2:** Effects of CCL21/CCR7 on MMP-2 and MMP-9 expression as represented via OD values.

Groups	MMP-2	MMP-9
Control	0.26 ± 0.03	0.17 ± 0.04
Anti-CCR7 Ab + CCL21 50 ng/ml	0.32 ± 0.05	0.20 ± 0.06
CCL21 50 ng/ml	0.43 ± 0.05	0.33 ± 0.05
CCL21 100 ng/ml	0.67 ± 0.07	0.41 ± 0.06
CCL21 200 ng/ml	0.80 ± 0.10	0.65 ± 0.08

Note: The OD values present Mean ± SD from 3 independent experiments.

### Effects of CCL21/CCR7 on apoptosis in T24 cells

**Fig 3:**
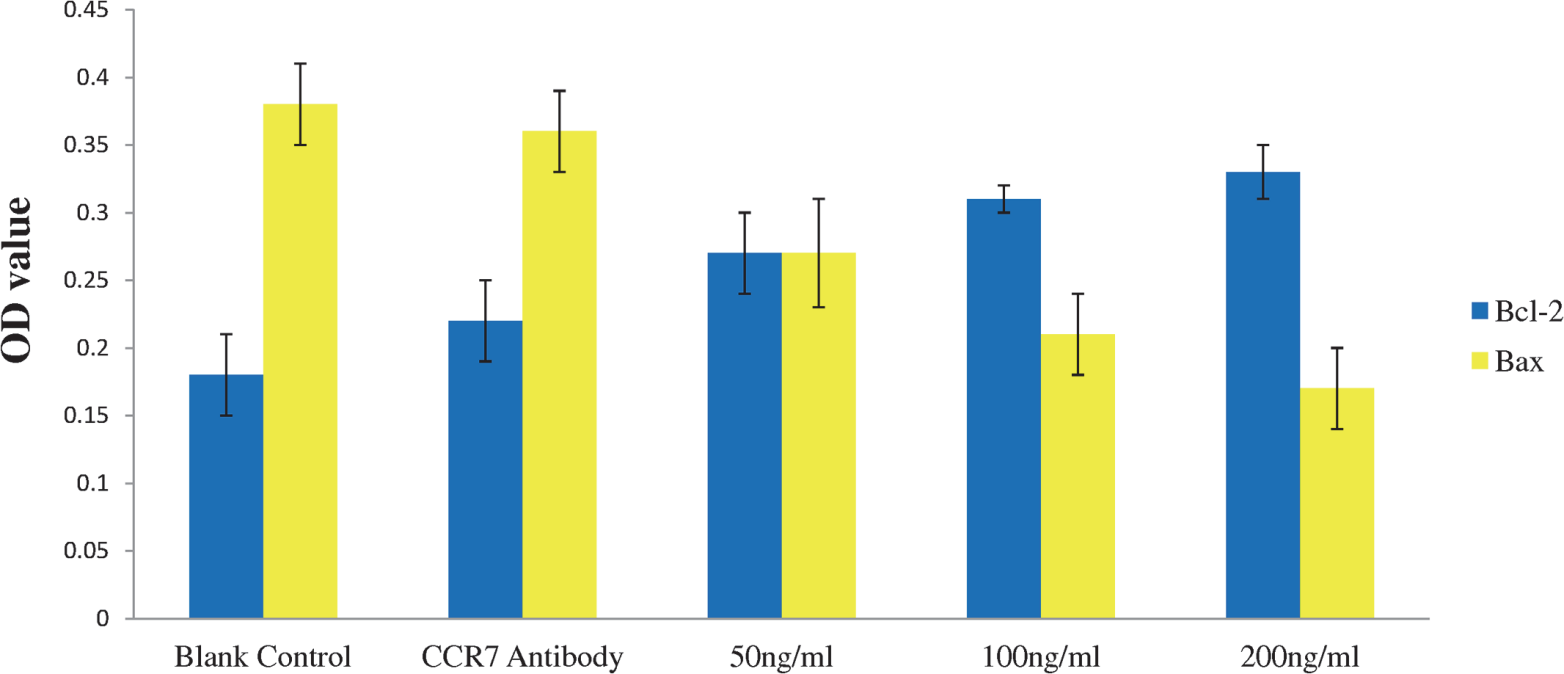
Effects of CCL21 with or without CCR7 antibody on the expressions of Bcl-2 and Bax. T24 cells were treated with vehicle control, CCR7 antibody 20 ng/ml plus CCL21 50 ng/ml, CCL21 50 ng/ml, CCL21 100 ng/ml, and CCL21 200 ng/ml. The values (OD) present Mean ± SD from three independent experiments.

Effects of CCL21/CCR7 on the expressions of Bcl-2 and Bax by Western blot are presented in Fig. 2 and the OD values are presented in Fig. 3. Protein expression of Bcl-21 follows an ascending trend while the expression of Bax follows a descending trend as the concentration of CCL21 increases. Significant differences were found in the expressions of Bcl-2 and Bax between CCL21 50 ng/ml group and CCL21 100 ng/ml group (p<0.05 for both) and between CCL21 50 ng/ml group and CCL21 200 ng/ml (p<0.05 for both) but not between CCL21 100 ng/ml group and CCL21 200ng/ml (p = 0.545 for Bcl-2 and p = 0.191 for Bax). Again, no difference was found between the control group and the antibody group for either Bcl-2 or Bax (p>0.05 for both).

Early and late apoptosis rates and total cell death rates after treatments are presented in Table 3. The rates of early apoptosis, late apoptosis and total cell death rates in the control group are 26.4 ± 5.0%, 37.5 ± 2.4%, and 63.9 ± 7.1%, respectively; whereas in the antibody group are 25.2 ± 3.8%, 35.6 ± 5.5%, and 60.8 ± 9.3%, respectively. No difference in apoptosis rates was found in each stage between the two groups (p > 0.05 for all). The rates of early apoptosis, late apoptosis and total cell death dropped after CCL21 treatments at 50, 100, and 200 ng/ml. Compared with control group, significant decreases in total cell apoptosis were found in CCL21 treatment groups (p < 0.001 for all three treatment groups); however, no significant difference was found among the three CCL21 treatment groups in total cell apoptosis (p > 0.05 for all). Compared with control group, significant decreases were also found in early cell apoptosis in CCL21 treatment groups (p = 0.008 for 200 ng/mL, 0.001 for 100 ng/mL, and 0.027 for 50 ng/mL); however, again, no significant difference was found among the three CCL21 treatment groups in early apoptosis (p > 0.05 for all).

**Table 3:** Antiapoptotic effects of CCL21/CCR7 on T24.

Groups		Early apoptosis (%)		Late apoptosis/necrosis (%)		Total cell death (%)	
Control		26.4 ± 5.0		37.5 ± 2.4		63.9 ± 7.1	
Anti-CCR7 Ab 50 ng/ml + CCL21 50 ng/ml		25.2 ± 3.8		35.6 ± 5.5		60.8 ± 9.3	
CCL21 50 ng/ml		19.6 ± 2.7		16.5 ± 2.1		36.1 ± 1.0	
CCL21 100 ng/ml		14.9 ± 1.8		17.8 ± 1.4		32.7 ± 1.5	
CCL21 200 ng/ml		17.7 ± 1.4		16.4 ± 3.3		34.1 ± 2.4	

Note: The values present Mean ± SD from 3 independent experiments.

## Discussion

The potential of cancer cells to metastasize depends on its interactions with homeostatic factors including chemokines which not only promote cancer cell growth and survival but also migration and invasion or metastasis. Previous studies have demonstrated that chemokines are involved in the progression and metastasis of various cancers[2, 4, 5]. CCR7 is involved in cancer metastasis to lymph nodes in various cancers[7–13]. CCL21 participates in the proliferation of CD4 T cells, kidney mesangial cells, and various cancer cells[19, 24–27]. Our study demonstrated for the first that CCL21/CCR7 could increase T24 cell proliferation (Table 1), migration and invasion (Fig. 1).

Chemokine concentration may be at least in part responsible for cancer development and metastasis[19, 26]. As a result, we used three different concentrations of CCL21 in the present study and found a concentration dependent manner in CCL21 effects on T24 cell proliferation, migration, and invasion. CCL21 may strongly increase T24 cell migration and invasion at high concentrations and thus facilitate bladder cancer metastasis. Our findings are consistent with these previous reports.

The interaction of chemokines and their receptors serve as the foundation for tumor cell motility and migration via triggering intracellular actin polymerization to create pseudopodia[28, 29]. Muller et al found that CCL21 at100 nM induced a transient 1.6-fold increase in intracellular flamentous actin (F-actin) in human breast cancer cells within 20 seconds and observed distinct pseudopodia formation after 20 min of stimulation with CCL21[19]. The results of present study with T24 cell migration and invasion are consistent0020with these previous reports. Significant decrease in T24 cell numbers was observed when CCR7 antibody was used together with CCL21 and it indicates that effect of CCL21 depends on the activity of CCR7. On the other word, CCL21 may activate CCR7 first and then affect T24 cell migration and invasion.

Molecules like chemokine CCL21 are involved in the metastasis of cancers not only via mediating the migration and invasion of cancer cells into tissues but also providing the required supportive microenvironments. In vivo, the migration of cancer cells is often slowed with lots of resistances, such as basement membrane and surrounding connective tissue[29, 30]. Therefore, to mimic the real in vivo condition, we utilized transwell chamber coated with matrigel as a physical barrier for the present study. MMP-2 and MMP-9 are two proteins which are closely associated with bladder cancer metastasis[31–33]. Cancer cell invasion may require the secretion of MMPs such as MMP-2 and MMP-9 to degrade the matrigel. In the present study, we found that CCL21 could increase the expression of MMP-2 and MMP-9 and thus enhance cell invasion (Fig. 2 and Table 2). MMPs play important roles in cancer cell invasion; but other factors such as interleukins and E-cadherin may also contribute to tumor progression and invasion [34–37]. Nonetheless, CCL21/CCR7 may facilitate cancer cell invasion via increasing MMP secretion and enhancing cell motility.

Using radiolabled techniques, researchers have found only a tiny portion of radiolabled cancer cells in microcirculations has the potential to metastasize[38]. The survival of cancer cells depends on their antiapoptotic abilities. When tumor cells leave its original tissues, they will lose some if not all the homeostatic factors which include chemokines for adhesion and support; thus the cells are prone to apoptosis. In the present study, the antiapoptotic ability of CCL21 inT24 cells were investigated using flow cytometry by annexin V-FITC/PI staining via ADM induced apoptosis. Phosphatidylserine (PS) can specifically bind annexin-V. PI, a nuclear staining agent, cannot cross normal cell membrane. In the early phase of apoptosis, PI nuclear staining cannot be detected as the cell membrane is intact. However, as cell undergoes apoptosis in the middle to late phases, cell membrane blebs and is no longer intact; thus nucleus can be detected through PI staining.

The result of the flow cytometry can be divided into four quadrants: the lower left quadrant with normal alive cells [Annexin-V (−), PI (−)], the upper right quadrant [Annexin-V (+), PI (+)] with late apoptotic or necrotic cells, the lower right quadrant [Annexin-V (+), PI (−)] with early apoptotic cells, and the upper left quadrant [Annexin-V (−), PI (+)] with small portions of cell debris due to mechanical injury and apoptosis. Thus, the analysis of apoptosis mainly depends on early apoptosis (lower right quadrant) and total cell death (upper right + lower right quadrants) in the present study. Our results demonstrated significant decrease of apoptosis and cell death with CCL21 treatment compared to the control (p < 0.01); however, no difference between CCR7 antibody plus CCL21 treatment and the control was found (p > 0.05). The results indicate that CCR7 antibody could antagonize the effect of CCL21 on apoptosis and cell death and thus confirm the antiapoptotic function of CCL21/CCR7. However, no difference of apoptosis was detected among the three treatment groups (p > 0.05). The possible cause could be that CCL21 at 50 ng/mL reached saturate condition thus higher concentrations could not further increase it effect.

Bcl-2 family is found to be closely related to cell apoptosis[39–41]. Bcl-2 and Bax are the two most important proteins in the Bcl-2 family with Bcl-2 inhibiting apoptosis while Bax promoting apoptosis. These two proteins are present in dimers, such as Bcl-2/Bcl-2 and Bax/Bax. Upregulation of Bcl-2 expression will lead to the additional Bcl-2 to combine with Bax forming Bcl2/Bax thus inhibit cell apoptosis; whereas with downregulation of the expression of Bcl-2, the quantity of the combined form of Bcl2/Bax will decrease and thus promote cell apoptosis. In the present study, we investigated the expression of Bcl-2 and Bax proteins in T24 cells in all experimental groups including the control. CCL21 could increase Bcl-2 protein expression but decrease Bax protein expression and the effects exhibited a CCL21 concentration dependent manner between 50 ng/mL and 100 ng/ml. Interestingly, no significant difference was found in Bcl-2 and Bax expressions between CCL21 at 100 ng/mL and 200 ng/ml CCL21 (Bcl-2: p = 0.545; Bax: p = 0.191); the cause may be that CCL21 reached to a certain concentration no higher than 100 ng/mL for its maximal anti-apoptotic effects (Fig. 3). Pretreatment of CCR7 antibody could almost completely reverse the effect of CCL21 on the expressions of Bcl-2 and Bax and apoptosis (Fig. 3 and Table 3). This further confirms the vital role of CCR7 on the effects of CCL21 on T24 cells.

## Conclusion

CCL21/CCR7 promoted T24 cell proliferation in a concentration dependent pattern and enhanced its migration and invasion; these effects may be related to the increased expression of MMP-2 and MMP-9. CCL21/CCR7 had antiapoptotic activities on T24 cells; these functions may be related to the regulation of Bcl-2 and Bax proteins. Our results indicate that CCL21/CCR7 may promote bladder cancer development and metastasis.
